# Medical or Surgical Treatment for Disabled Nurses

**Published:** 1920-03-13

**Authors:** 


### Medical or Surgical Treatment for Disabled Nurses.

The Minister of Pensions has now entrusted to the Commissioners of Medical Services in the various regions of the Ministry the responsibility of obtaining for disabled officers and nurses the medical or surgical treatment to which they are entitled under the Royal Warrant. A discharged officer or nurse who is claiming retired pay or pension on the ground that he or she is suffering from a disability which is either attributable to or aggravated by service, and who is in need of treatment for the disability, should also make application to the Commissioner of Medical Services at the Regional Office, with a view to the necessary treatment being provided. In the event of officers or nurses making their own arrangements for treatment without the prior approval of the Commissioner of Medical Services of the Ministry of Pensions, it may not be possible to refund to them any of the expenses thereby incurred.

The following is a list of the addresses of the Regional Commissioners and the counties comprised in each region, but the Local War Pensions Committee will supply any officer or nurse with the address of the appropriate regional office upon request.

| Region. | Counties. | Addresses. |
|---|---|---|
| Scotland. | All Scotland. | Adelphi Hotel, Cockburn Street, Edinburgh. |
| Northern. | Northumberland. Durham. Cumberland. | 14 Clayton Street West, Newcastle-on-Tyne. |
| North Western. | Lancashire. Cheshire. Westmorland. Isle of Man. | 13 Piccadilly, Manchester. |
| Yorkshire. | Yorkshire. | 7 Board Lane, Leeds. |
| Wales. | All Wales and Monmouthshire. | Angel Building, Cardiff. |
| West Midlands. | Staffordshire. Shropshire. Warwickshire. Worcestershire. Herefordshire. | Bethany Buildings, Loveday Street, Birmingham. |
| East Midlands. | Leicestershire. Lincolnshire. Nottinghamshire. Derbyshire. Northamptonshire. Rutlandshire. | Black's Building, Stoney Street, Nottingham. |
| South Western. | Gloucestershire. Wiltshire. Dorsetshire. Somersetshire. Devonshire. Cornwall. | Clifton Down Buildings, Bristol. |
| Eastern. | Norfolk. Suffolk. Cambridgeshire. Huntingdonshire. Essex. Bedfordshire. Hertfordshire. Buckinghamshire. Oxfordshire. Berkshire. | 80 Westbourne Terrace, W. 2. |
| London. | Metropolitan Area. | Crown Agents' Annexe, Westminster House, Millbank, S.W. 1. |
| South Eastern. | Kent. Surrey. Sussex. Hampshire. Isle of Wight. Channel Isles. | 48 Grosvenor Gardens, S.W. 1 |
| Ulster. | Ulster. | Grand Central Hotel, Belfast. |
| Ireland, South. | Munster. Leinster. Connaught. | Dunlop House, Abbey Street, Dublin. |

Any communication with regard to the award of retired pay or pension should be addressed to the Officers' Award Branch, Cromwell House Annexe, Millbank, S.W. 1.

